# Transcriptomic and metabolomic analysis unveils a negative effect of glutathione metabolism on laccase activity in *Cerrena unicolor* 87613

**DOI:** 10.1128/spectrum.03405-23

**Published:** 2024-01-17

**Authors:** Long-Bin Zhang, Ting-Ting Qiu, Xiu-Gen Qiu, Wu-Wei-Jie Yang, Xiu-Yun Ye, Chun Meng

**Affiliations:** 1 The Key Laboratory of Marine Enzyme Engineering of Fujian Province, Fuzhou University, Fujian, China; 2 College of Biological Science and Engineering, Fuzhou University, Fujian, China; Universidade de Brasilia, Brasília, Brazil

**Keywords:** *Cerrena unicolor*, laccase activity, glutathione metabolism, transcriptome, metabolome

## Abstract

The white rot fungus *Cerrena unicolor* 87613 has been previously shown to be a promising resource in laccase production, an enzyme with significant biotechnological applications. Conventional methods face technical challenges in improving laccase activity. Attempts are still being made to develop novel approaches for further enhancing laccase activity. This study aimed to understand the regulation of laccase activity in *C. unicolor* 87613 for a better exploration of the novel approach. Transcriptomic and metabolomic analyses were performed to identify key genes and metabolites involved in extracellular laccase activity. The findings indicated a strong correlation between the glutathione metabolism pathway and laccase activity. Subsequently, experimental verifications were conducted by manipulating the pathway using chemical approaches. The additive reduced glutathione (GSH) dose-dependently repressed laccase activity, while the GSH inhibitors (APR-246) and reactive oxygen species (ROS) inducer (H_2_O_2_) enhanced laccase activity. Changes in GSH levels could determine the intracellular redox homeostasis in interaction with ROS and partially affect the expression level of laccase genes in *C. unicolor* 87613 in turn. In addition, GSH synthetase was found to mediate GSH abundance in a feedback loop. This study suggests that laccase activity is negatively influenced by GSH metabolism and provides a theoretical basis for a novel strategy to enhance laccase activity by reprogramming glutathione metabolism at a specific cultivation stage.

**IMPORTANCE:**

The production of laccase activity is limited by various conventional approaches, such as heterologous expression, strain screening, and optimization of incubation conditions. There is an urgent need for a new strategy to meet industrial requirements more effectively. In this study, we conducted a comprehensive analysis of the transcriptome and metabolome of *Cerrena unicolor* 87613. For the first time, we discovered a negative role played by reduced glutathione (GSH) and its metabolic pathway in influencing extracellular laccase activity. Furthermore, we identified a feedback loop involving GSH, GSH synthetase gene, and GSH synthetase within this metabolic pathway. These deductions were confirmed through experimental investigations. These findings not only advanced our understanding of laccase activity regulation in its natural producer but also provide a theoretical foundation for a strategy to enhance laccase activity by reprogramming glutathione metabolism at a specific cultivation stage.

## INTRODUCTION

Laccase (EC1.10.3.2) is a blue multicopper-containing phenol oxidase that can oxidize a variety of substrates using oxygen as an electron acceptor (1). In the last decades, laccase has gained considerable attention as a green biocatalyst for industrial biotechnology applications (2, 3). For example, laccase was used to stabilize the color or flavor of wines through the elimination of specific phenols and polyphenols (4, 5). Laccase also has the potential for pollutant degradation (6), such as dyes (7) and pharmaceuticals (8). In the biofuel production process, laccase-mediated delignification improved the utilization efficiency of lignocellulosic biomass (9, 10). Additionally, this blue-cooper enzyme could function as a therapeutic agent with antibacterial and/or antioxidant properties as well (11, 12). Although laccase offers a wide range of biotechnology applications, the production of its activity is insufficient to meet demand. Engineering strategies are urgently needed to further enhance its acquisition.

Heterologous expression of laccase has been used to improve its acquisition. However, the laccase production achieved through heterologous expression is generally lower compared to that of native sources. For instance, the activity of hetero-expressed *Cerrena* sp. laccase showed 20–50 times lower than those of native sources (around 121.7–333.2 U/mL) (13–15). A similar phenomenon was also observed in the hetero-expression of *Trametes versicolor* laccase (16–18). These are due to the fact that laccase is a glycoprotein and the correct pattern of its glycosylation is not maintained in a non-native expression system (2). As a result, laccases produced by non-native sources might be sensitive to the host’s proteases and thus be easily degraded, leading to low activity (19, 20). Therefore, the hetero-expressing strategy is not as efficient as we expect to fulfill the industrial requirements of laccase. Alternative methods to improve the acquisition of laccase activity were considered, including screening for high-laccase-producing sources and optimizing cultivation conditions. Laccase could be produced by fungi, bacteria, plants, and even insects (21). Fungi, especially white rot fungi, show a predominant role in laccase production (22). Among those, *Cerrena unicolor* has been targeted and intensively studied as a promising source of fungal laccase (8). Thereafter, optimization of incubation conditions, such as carbon/nitrogen source, initial pH, incubation temperature, shaking speed, and metal ion concentration, was further conducted to enhance the laccase activity (14, 23). Different cultivation methods, including solid/liquid-state cultivation and co-cultivation of *C. unicolor* with other fungal species, have also been explored (22, 24). Additionally, supplementing xenobiotics like wheat bran or other lignocellulosic substrates can increase laccase activity (18, 25). However, these strategies have upper limitations in the production of laccase activity and take additional costs as well. Hence, current efforts are focused on exploring new strategies to reduce costs and further break the upper limitations of laccase activity produced by native sources.

Metabolic reprogramming is widely used in the treatment of cancer and other diseases (26) but rarely applied in the improvement of enzyme production in microorganisms. Recently, studies have shown promising effects of metabolic reprogramming in reversing antibiotic resistance of bacteria (27–29). These reports inspired us to explore the use of metabolic reprogramming to boost the productivity of laccase from its native sources. However, a basic concept of the metabolic pathway controlling laccase activity for precise metabolic reprogramming is missing. In this study, we examined a strain *C. unicolor* 87613, which exhibited a higher laccase (415 U/mL) compared to other *Cerrena* strains (30). The high baseline level of laccase production of this particular strain provided an advantageous foundation for our investigation. Subsequently, this study presents evidence of the negative impact of the glutathione metabolism pathway on the regulation of laccase activity, based on the analysis of transcriptome and metabolome. To the best of our knowledge, it is the first time to unveil the metabolite-regulatory mechanism of laccase activity in *C. unicolor* or other Basidiomycetes. The primary objective of this study is to gain metabolic insights into the regulation of laccase activity and establish a theoretical foundation for the development of engineered fungi capable of producing laccase more efficiently.

## RESULTS AND DISCUSSION

### Selection of different laccase-production periods for omics sequencing

Due to the significant demand for laccase in various industrial biotechnology applications (2), there is a need to improve its current production. In our previous study, a white rot fungus *C. unicolor* 87613 was reported with a high production level of extracellular laccase (laccase, 415 U/mL) (30). This is considerably higher than other *Cerrena* strains, which have laccase activities ranging from 121.7 to 333.2 U/mL in shake-flask experiments (8, 14, 15). Therefore, *C. unicolor* 87613 shows promise as a source for laccase production.

To gain a better understanding of the regulatory mechanism of laccase production in *C. unicolor* 87613, we employed RNA sequencing and LC-MS/MS technology to analyze the intracellular transcriptome and metabolome profiles. Notably, our observation of the laccase-producing pattern during 12-day cultivation revealed that laccase activity began to increase on cultivation day 2 (Cd-2), reached the peak of laccase activity at Cd-6, and then declined to a low of 307 U/mL by Cd-10 (Fig. S1). Interestingly, the laccase activity increased again from Cd-10 to Cd-12, possibly due to a concentration effect resulting from the reduction of cultivation broth volume as the mycelia grew. Consequently, we selected replicated cultures from Cd-6 (the period of high laccase activity) and Cd-10 (the period of low laccase activity) for transcriptomic and metabolomic investigations.

### Targeting glutathione metabolism pathway by combination analysis of two omics

**Fig 1:**
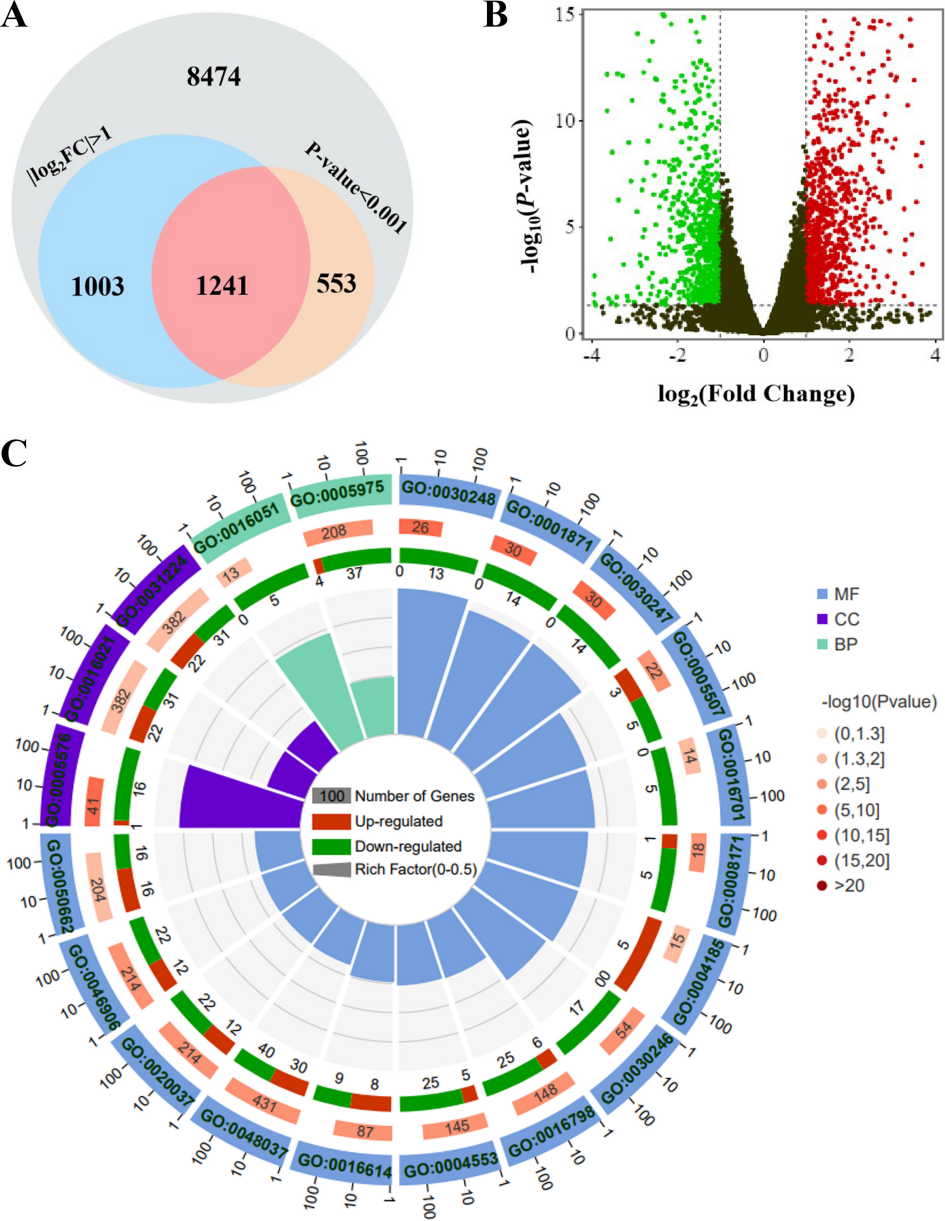
Changes of transcriptomic profiles in *C. unicolor* 87613 at cultivation day 6 (Cd-6) versus those at cultivation day 10 (Cd-10). (**A**) 1,241 DEGs were identified from 11,271 predicted genes. (**B**) The volcano map of the transcription profile of all predicted genes at Cd-6 compared to that at Cd-10. The red dots indicate genes with values of log_2_ (Cd-6/Cd-10 ratio) ≥ 1 and of *P*-value < 0.001, while the green dots present genes with values of log_2_ (Cd-6/Cd-10 ratio) ≤ −1 and of *P*-value < 0.001. (**C**) The circle map of GO function enriched by DEGs. The outer circle represented the GO clusters enriched by DEGs, in which MF, CC, and BP clusters were marked in blue, purple, and green, respectively; the secondary outer circle layer indicated the number of total sequencing genes enriched in each subcategory, and the more genes were enriched, the longer the strip presented, in addition, the strip color represented −log_10_(*P*-value), the darker the color was, the higher the significance was; the secondary inner circle layer exhibited the number of up-regulated (red) or down-regulated (green) DEGs enriched in each subcategory, respectively; the inner circle represented the RichFactor of each subcategory, which referred to the proportion of enriched DEGs in all sequencing genes, and every cell represented for 0.1 value of RichFactor.

RNA sequencing was first performed to unveil the altered transcription profile at Cd-6 versus Cd-10. As presented in Table S1, the values of Q20 (>97%), Q30 (>93%), and the percentage of mapped reads to total reads (around 95%) validated the reliability of these transcriptomic data. A total of 11,271 transcripts were detected (Fig. 1A), which is close to those in *C. unicolor* FCL139 with 12,966 predicted genes (31). Among those, 1,241 genes were identified as differentially expressed genes (DEGs) according to their values of |log_2_ (Cd-6/Cd-10 ratio)| ≥ 1 and of *P*-value < 0.001 (Fig. 1A). Seven hundred and ten up-regulated genes and 531 down-regulated genes were recognized at Cd-6 versus those at Cd-10 (Fig. 1B). Among those, the top three up-regulated DEGs included short-chain dehydrogenase, *CuLac15*, and oxidoreductase, while the most down-regulated DEGs were alcohol oxidase, cellulase, and cytochrome. All the DEGs were then categorized into Biological Process (BP), Cellular Component (CC), and Molecular Function (MF) based on the Gene Ontology (GO) database (Fig. 1C; Table S2). The enriched BP and MF terms were related to metabolic processes and/or enzymatic activities. All of these GO terms were considered to be repressed as the counts of their down-regulated DEGs exceeded the counts of their up-regulated DEGs. Based on the quantity difference between up-regulated and down-regulated DEGs in each term, we found that the “carbohydrate metabolic process” in BP terms was mostly repressed, followed by “hydrolase activity” in MF terms (Table S2). In addition, 10 Kyoto Encyclopedia of Genes and Genomes (KEGG) pathways were enriched by the DEGs (Table S3). Six of them were transcriptionally repressed during the Cd-6 period, while others were facilitated. Notably, both GO and KEGG analyses primarily focused on metabolic processes. These findings suggest that metabolism plays a regulatory role in fungal phenotypes. For example, the other transcriptome study of *C. unicolor* FC139 demonstrated that metabolism was a primary response to different light conditions (31).

**Fig 2:**
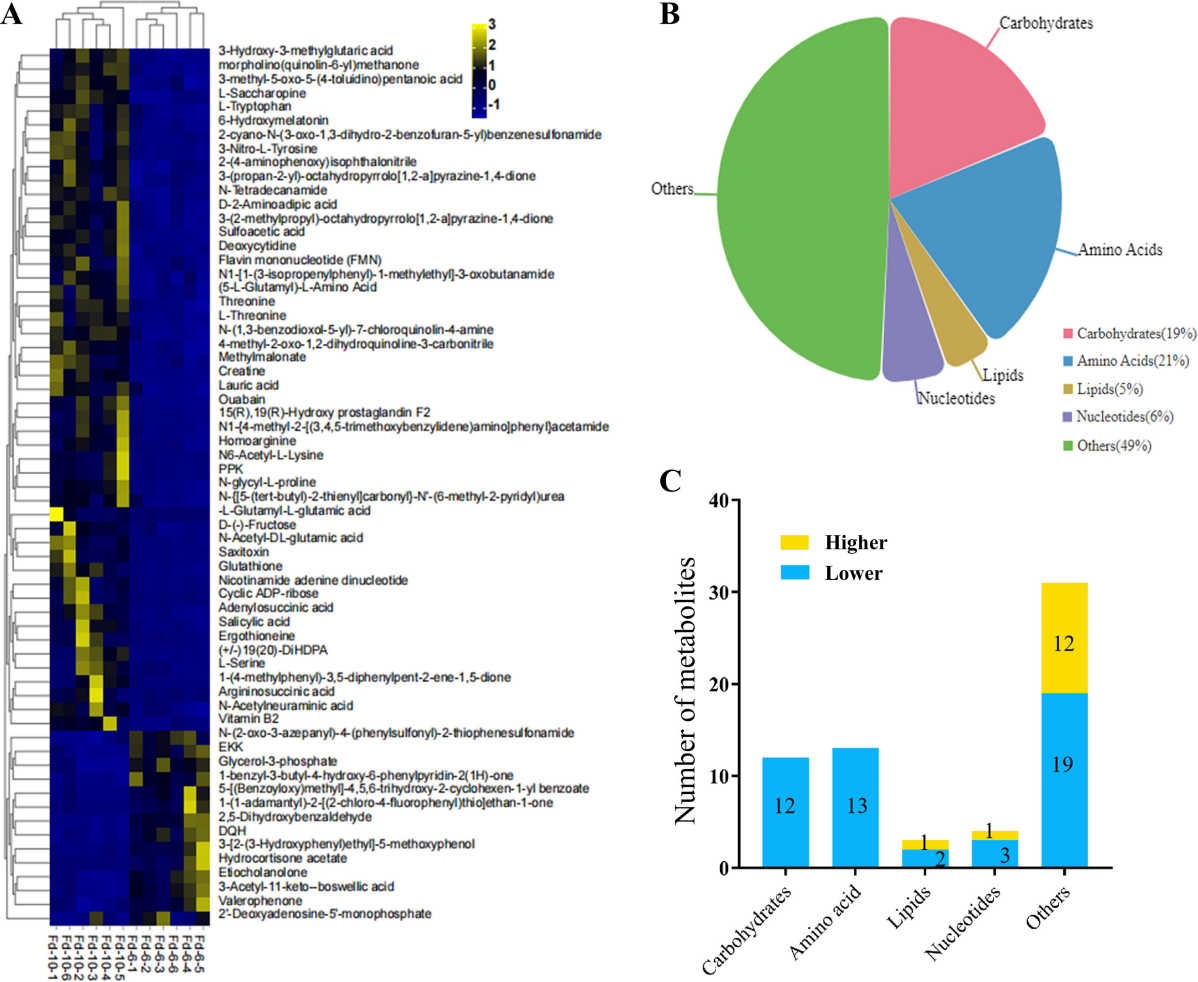
Changes of metabolomic profiles in *C. unicolor* 87613 at Cd-6 versus those at Cd-10. (**A**) The heatmap of the metabolomic profile of all DAMs at Cd-6 compared to that at Cd-10. (**B**) Categories of metabolites exhibiting differential abundances. (**C**) Number of metabolites with differential abundances.

The transcriptomic analysis motivated us to further investigate the changes in metabolome between two different cultivation periods. Thereafter, we carried out LC-MS/MS analysis on six replicates from each Cd-6 and Cd-10 period. As a result, we identified 1,345 metabolites, among which 63 metabolites were defined as differentially abundant metabolites (DAMs) based on their values of |log_2_ (Cd-6/Cd-10 ratio)| ≥ 1 and of *P*-value < 0.05. Of these DAMs, 14 were increased and 49 were decreased at the Cd-6 period compared to the Cd-10 period (Fig. 2A). These DAMs fell into different categories, including carbohydrates (12), amino acids (13), lipids (3), nucleotides (4), and others (31) (Fig. 2B). Notably, some important bioactive substances like reduced glutathione (GSH), phenolic compounds, and terpenoids (32, 33) were found among these DAMs. Most of them showed lower abundance at the Cd-6 period compared to the Cd-10 period (Fig. 2C). We also used MetaboAnalyst 5.0 software (34) to enrich the metabolic pathways. As a result, the top 10 significantly enriched pathways were mainly involved in amino acid metabolism, glutathione metabolism, fatty acid metabolism, and saccharide metabolism (Fig. S2A). Importantly, these pathways were globally attenuated at the Cd-6 period due to the decreased abundance levels of their components (Fig. S2B). These findings suggest that the reduction of laccase production from Cd-6 to Cd-10 period in *C. unicolor* 87613 might be attributed to the increased abundance of these pathways.

**Fig 3:**
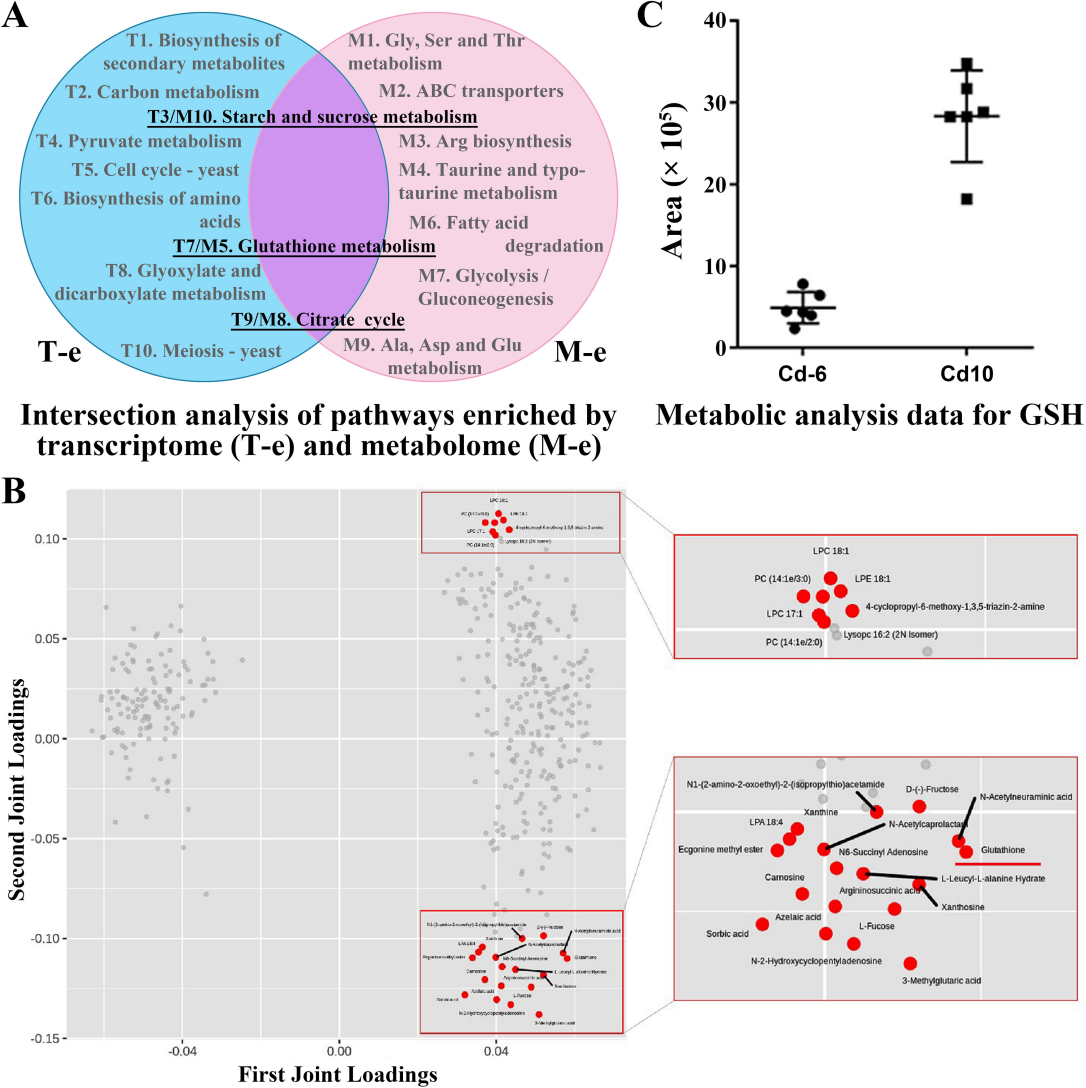
The glutathione metabolism was targeted by the combination analysis of transcriptomic and metabolomic data. (**A**) Three pathways of starch and sucrose metabolism, glutathione metabolism, and citrate cycle co-enriched by pathway analysis of dual omics data. (**B**) Glutathione targeted from 25 DAMs with the strongest correlation with transcriptome data, using dual omics correlation analysis by O2PLS. (**C**) The abundances of reduced glutathione at Cd-6 and Cd-10, respectively.

To further focus on the key metabolic pathway associated with laccase activity, we performed a combination analysis of the two omics. The analysis revealed three co-enriched pathways, namely “Starch and sucrose metabolism,” “Glutathione metabolism,” and “Citrate cycle” (Fig. 3A). We then used O2PLS software to estimate the correlation between the two omics data. Consequently, the analysis identified several potential factors (marked in red), among which the glutathione showed a significantly lower abundance at the Cd-6 period compared to the Cd-10 period (Fig. 3B and C). These results indicate that glutathione and its metabolic process might negatively regulate laccase production in *C. unicolor* 87613. As far as we know, none of study have reported this regulatory mechanism of laccase production.

### Variations of glutathione metabolism and related intracellular environment between Cd-6 and Cd-10 period

**Fig 4:**
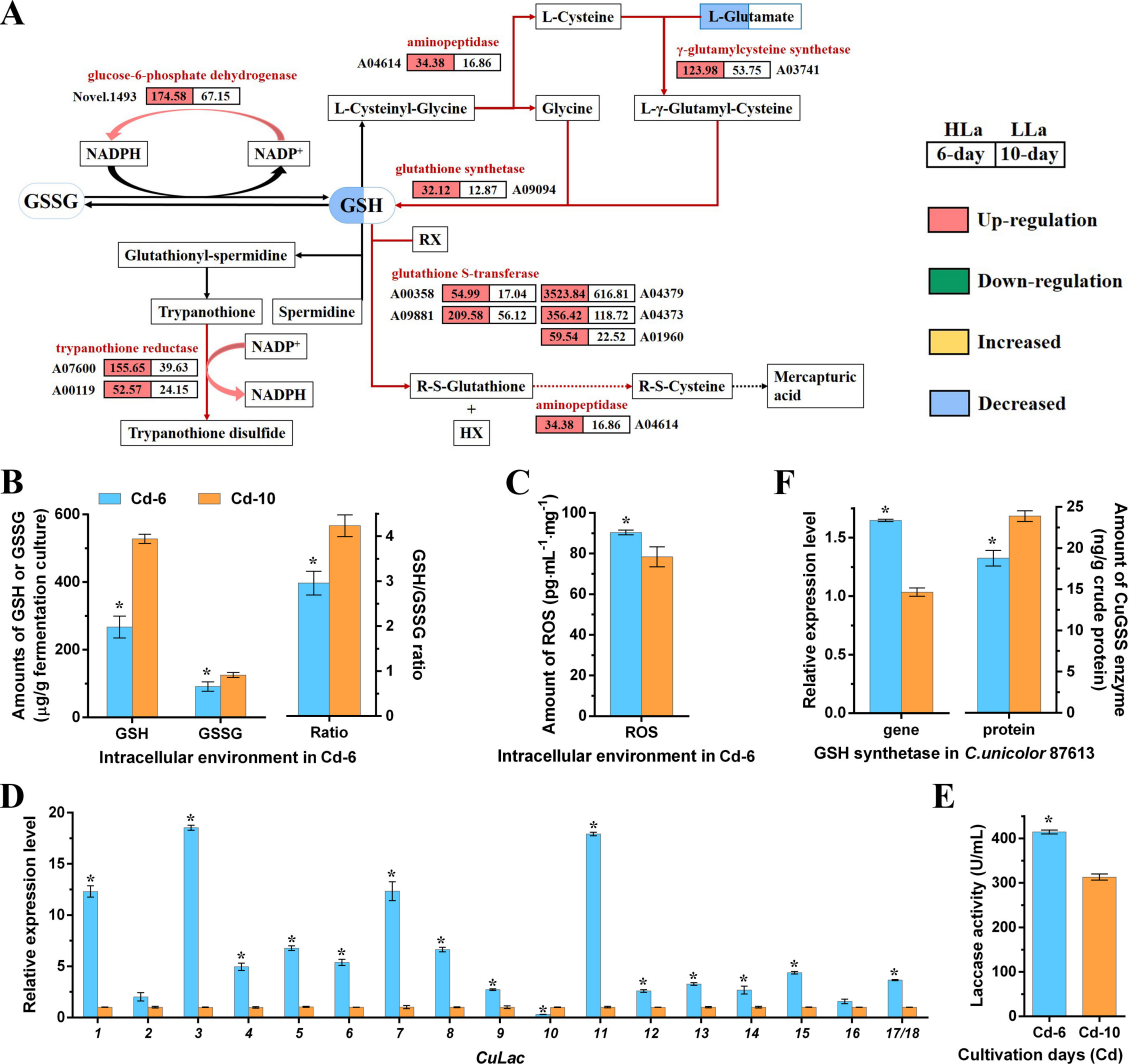
Comparison of GSH metabolism, ROS level, and Cu*Lac*s expression between cultivation day 6 (Cd-6) and day 10 (Cd-10) in *C. unicolor* 87613. (**A**) The changes in gene expression and metabolite abundances in the GSH metabolism pathway between two different periods. (**B**) The amounts of reduced and oxidized glutathione (GSH and GSSG) and their ratios at two periods. (**C**) The ROS levels at Cd-6 and Cd-10 periods. (**D**) Relative expression levels of each Cu*Lac* gene at Cd-6 versus those at Cd-10. (**E**) Laccase activity at Cd-6 versus that at Cd-10. (**F**) Relative expression level of Cu*GSS* gene and its protein amount at Cd-6 versus those at Cd-10, respectively. *Asterisked bars* in each graph differ significantly from those unmarked (Tukey’s honestly significant difference (HSD), *P* < 0.05).

To support our deduction, we initially examined the intracellular changes during periods of high (Cd-6) and low (Cd-10) laccase activity. Based on KEGG analysis and previous reports (35, 36), we illustrated the glutathione metabolism pathway and identified the DEGs and DAMs within this pathway (Fig. 4A). Eleven genes involved in glutathione metabolism were found to be up-regulated at the Cd-6 period compared to the Cd-10 period (Fig. 4A; Table S4). Interestingly, despite the transcriptional activation of glutathione metabolic enzymes, the levels of both GSH and glutamate were decreased (Fig. 4A). Particularly, GSH is a tripeptide that plays a role in various cellular processes (37). Early studies have suggested a feedback effect of GSH on its own synthesis by inhibiting glutamate cysteine ligase and γ-glutamylcysteine synthetase (35, 38, 39). This negative correlation between GSH and its synthesis process might explain the opposite changes observed in GSH abundance compared to the transcription levels of related enzymes.

Experimental evidence has been conducted to further confirm the omics’ findings. Based on DNTB-circular-reaction measurement, the levels of GSH at Cd-6 were 49% lower compared to those at Cd-10 (Fig. 4B). The oxidized form of glutathione (GSSG) (40) was also 27% lower at Cd-6, resulting in a lower GSH/GSSG ratio (Fig. 4B). Additionally, the intracellular level of reactive oxygen species (ROS) was 15% higher at Cd-6 than those at Cd-10 (Fig. 4C). Regarding the fact that GSH and ROS play counter-balance roles in regulating intracellular redox state (41), these findings of lower GSH/GSSG ratio and higher ROS level suggest a more oxidizing environment in *C. unicolor* 87613 mycelia at Cd-6 period (41, 42). Previous studies have shown that oxidative stress could enhance the expression of fungal laccase (43, 44). Accordingly, we found a globally activated expression of 14 laccase (Cu*LAC*) genes under a more oxidizing environment at Cd-6 period compare to Cd-10 period (Fig. 4D), which might contribute to the elevation of laccase activity (Fig. 4E).

Furthermore, the transcription level of GSH synthetase (Cu*GSS*), catalyzing the final step of GSH synthesis (45), was accordantly higher at Cd-6 (Fig. 4F). However, the amount of CuGSS enzyme was decreased at Cd-6 (Fig. 4F), possibly due to a delay in the translation process. This discrepancy between gene and enzyme levels might be attributed to the complexity of gene expression regulation (46). On the other side, GSS enzymes with less amount might reduce the efficiency of GSH synthesis (47), which was in accordance with our results. Overall, these findings suggest that GSH synthesis might be regulated by a feedback loop involving GSH, Cu*GSS* genes, and CuGSS enzyme; changes in GSH synthesis could influence the balance between GSH/GSSG and ROS, leading to oxidative stress and then the activation of laccase expression.

### Determination of the negative effect of exogenous GSH on laccase activity

**Fig 5:**
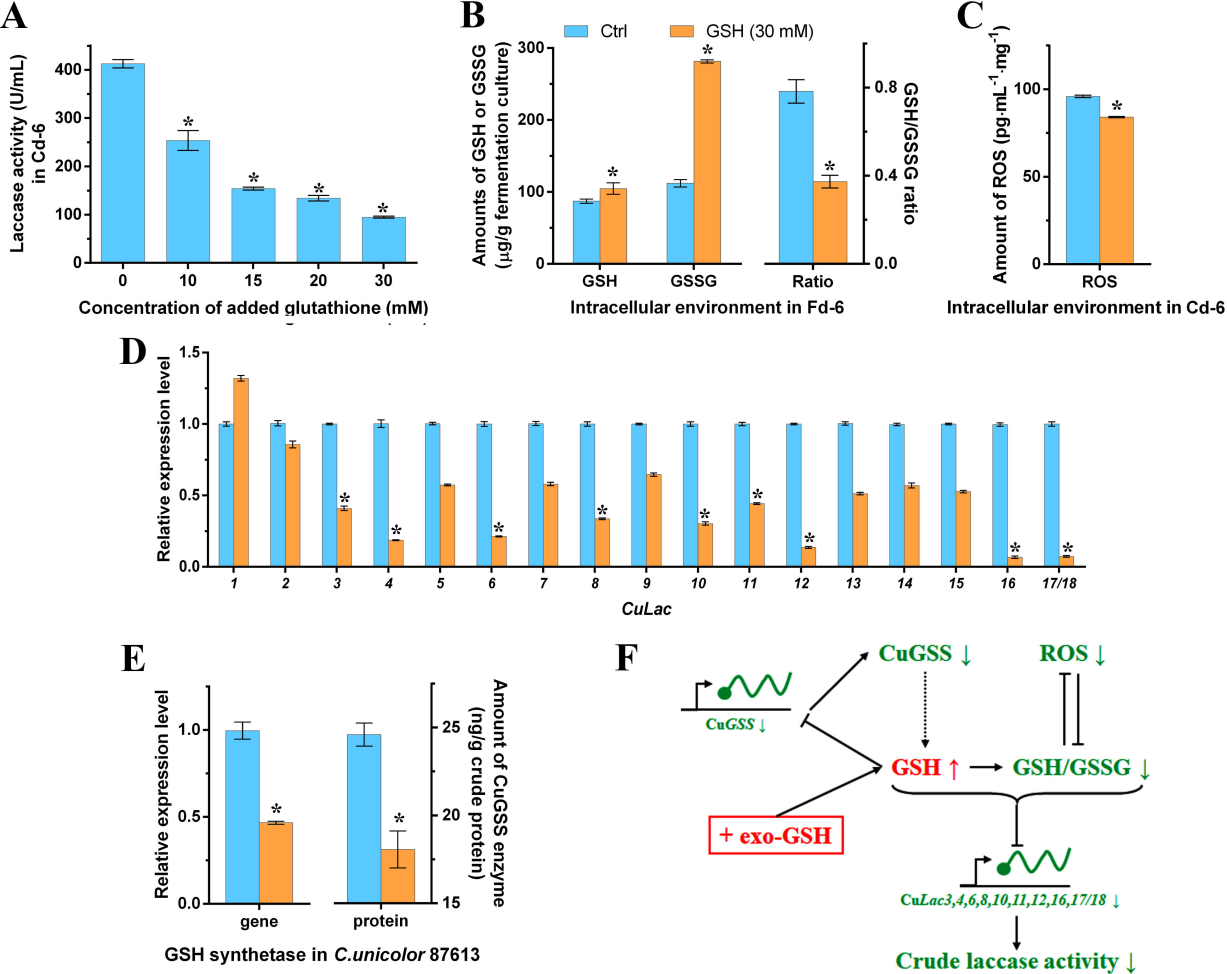
Negative effect of additive GSH on the levels of GSH synthetase (CuGSS), Cu*Lac*s, and ROS. (**A**) The dose-dependent repression of laccase activity by additive GSH in *C. unicolor* 87613. (**B**) The amounts of GSH, GSSG, and their ratio in GSH-treatment (30 mM) and control groups, respectively. (**C**) The ROS levels in GSH-treatment and control groups. (**D**) Relative expression levels of the Cu*Lac* gene family under the GSH-treatment versus those under the control. (**E**) The relative expression level of Cu*GSS* gene and its protein amount in GSH-treatment and control groups, respectively. (**F**) Illustration of the potential mechanism of additive GSH regulating laccase activity through the changes of the expression of Cu*GSS* and Cu*Lac* genes, and intracellular redox homeostasis. *Asterisked bars* in each graph differ significantly from those unmarked (Tukey’s HSD, *P* < 0.05).

To demonstrate the presumption mentioned above, we introduced external GSH into the cultivation media on Cd-4 and measured various intracellular parameters after a 2-day treatment (Cd-6). As shown in Fig. 5A, laccase activity decreased as the concentration of additive GSH increased, suggesting a dose-dependent negative regulation of laccase production by GSH. Previous studies have explored the effects of phenolic compounds (such as ferulic acid and veratric acid) and metal ions (such as Cu^2+^, Mn^2+^, and Cd^2+^) on fungal laccase production (23, 48–50). This is the first evidence demonstrating the inhibitory effect of oligopeptide GSH on fungal laccase activity.

To further investigate the impact of GSH, we compared the intracellular parameters of mycelia in the GSH-treatment (30 mM, the highest solubility of GSH at room temperature) with those in the control group. Surprisingly, the GSH-treatment group showed only a 17% increase in intracellular GSH content, but a 60% increase in GSSG content compared to the control group (Fig. 5B). A previous study indicated that a large amount of exogenous GSH could maintain a highly reducing intracellular environment by rapidly converting into GSSG (51). Hence, despite the lower GSH/GSSG ratio (reduced by 52%) caused by unusually high levels of GSSG, the intracellular environment remained more reductive in the GSH-treatment group. This deduction was further supported by a 12% reduction in ROS levels observed in the GSH-treatment group (Fig. 5C). Furthermore, we found that the transcription levels of most Cu*Lac* genes were down-regulated except for Cu*Lac1* (Fig. 5D). According to our previous discoveries and other studies (43), this down-regulation of Cu*Lac*s expression could be attributed to the high reducing intracellular environment. In addition, their down-regulation might contribute to the decrease in laccase production. Moreover, the additive GSH led to a 53% reduction in the transcription level and a 27% decrease in the enzyme amount of CuGSS (Fig. 5E). Previous studies have reported the inhibitory effect of GSH on glutamate cysteine ligase and γ-glutamylcysteine synthetase (35, 38, 39). In our current study, we additionally revealed its feedback effect on Cu*GSS* expression. In conclusion, the additive exogenous GSH might inhibit Cu*GSS* expression, thereby repressing the GSH metabolic process. Meanwhile, the additive GSH shifts the intracellular environment toward a more reduced state through an imbalanced GSH/ROS interaction, ultimately leading to reduced expression of Cu*Lac* genes and then the laccase activity produced by *C. unicolor* 87613 (Fig. 5F).

### Assaying the positive effect of GSH inhibitor APR-246 on regulating laccase activity

**Fig 6:**
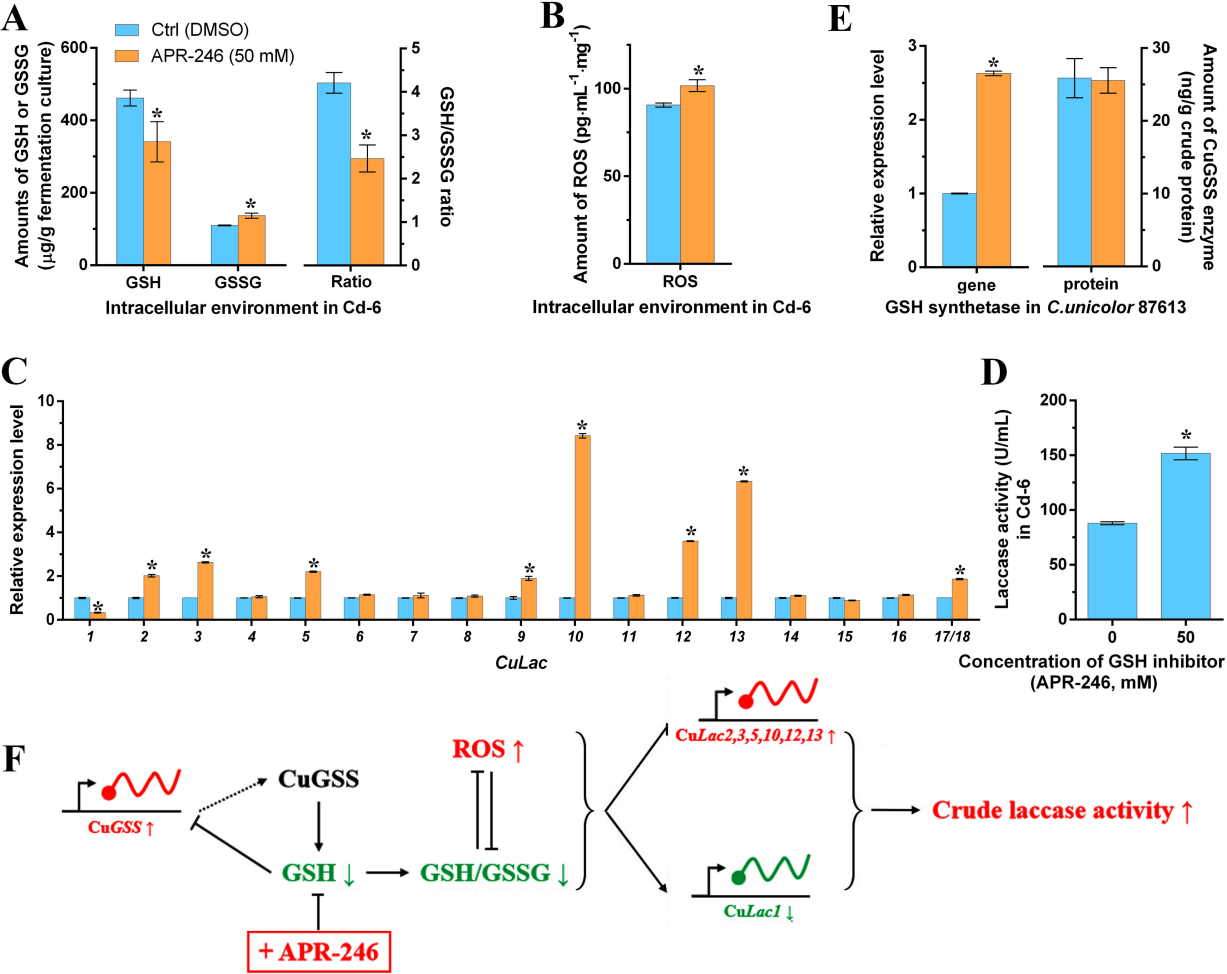
Positive effect of additive GSH inhibitor (APR-246) on CuGSS, Cu*Lac*s, and intracellular redox homeostasis. (**A**) The amounts of GSH, GSSG, and their ratio in APR-246-treatment (50 mM) and control (DMSO) groups, respectively. (**B**) The ROS levels in APR-246-treatment and control groups. (**C**) Relative expression levels of the Cu*Lac* gene family under the APR-246-treatment versus those under the control. (**D**) Laccase activity under the APR-246-treatment versus that under the control. (**E**) The relative expression level of Cu*GSS* gene and its protein amount in APR-246-treatment and control groups, respectively. (**F**) Illustration of the potential mechanism of additive APR-246 regulating laccase activity through the changes of the expression of Cu*GSS* and Cu*Lac* genes, and intracellular redox homeostasis. *Asterisked bars* in each graph differ significantly from those unmarked (Tukey’s HSD, *P* < 0.05).

To fully validate our deduction, we used a GSH inhibitor (APR-246) (52) to suppress the GSH synthesis during laccase cultivation in *C. unicolor* 87613. As illustrated in Fig. 6A, APR-246-treatment resulted in a 26% loss of GSH compared to the control group. Additionally, the abundance of GSSG was increased by 25%, leading to a 41% reduction in the GSH/GSSG ratio in the APR-246-treatment group (Fig. 6A). On the contrary, the ROS level was increased by 12% (Fig. 6B). The imbalance between GSH/GSSG and ROS induced more oxidative stress in the APR-246-treated group. As expected, the reduction of GSH by APR-246-treatment caused an up-regulation of the transcription levels of Cu*Lac2*,*3*,*5*,*9*,*10*,*12*,*13*,*17*/*18*, which led to a 73% enhancement in total laccase activity in turn (Fig. 6C and D). The promoter region of many laccase genes contains the antioxidant response element (ARE) and stress-responsive element (STRE), which are responsible for responding to oxidative stress (49, 53). Our previous study has shown that only Cu*Lac3*,*6*,*8*,*9*,*10*,*12–18* contained ARE and/or STRE (30), suggesting that GSH might regulate Cu*Lac2* and Cu*Lac5* through a pathway independent of oxidative stress. In addition, it was important to note that the APR-246 solvent DMSO somehow showed toxicity to fungal growth, which depressed the production of laccase. Nevertheless, the strains treated with APR-246 still performed higher laccase production as compared to those treated with DMSO (control). Furthermore, although there was no significant change in the amount of CuGSS enzyme, the expression of the Cu*GSS* gene was still increased by 2.6 times due to altered intracellular GSH levels (Fig. 6E). Based on these findings, we propose a potential regulatory mechanism of laccase activity via the GSH metabolism pathway (Fig. 6F): APR-246-mediated suppression of GSH facilitates the GSH metabolism pathway, leading to changes in the intracellular environment toward an oxidative state through negative interaction with ROS; this, in turn, activates the transcription of Cu*Lac* genes, resulting in increased laccase production.

### Effect of H_2_O_2_-induced oxidative stress on the laccase activity

**Fig 7:**
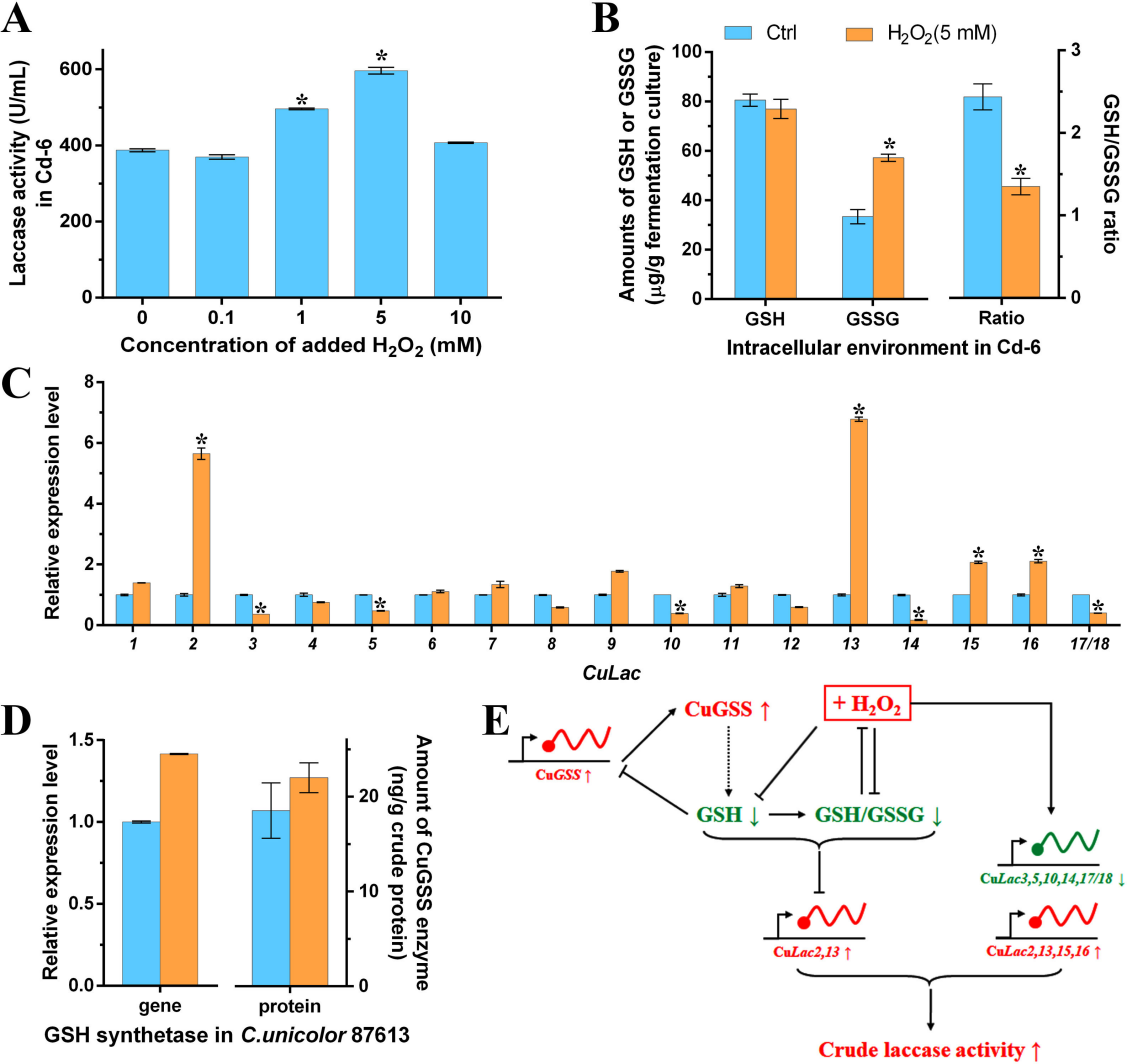
The influence of ROS inducer H_2_O_2_ on CuGSS, Cu*Lac*s, and intracellular redox homeostasis. (**A**) The dose-dependent repression of laccase activity by H_2_O_2_ (within a low concentration range). (**B**) The amounts of GSH, GSSG, and their ratio in H_2_O_2_-treatment (5 mM) and control groups, respectively. (**C**) Relative expression levels of the Cu*Lac* gene family under the H_2_O_2_-treatment versus those under the control. (**D**) The relative expression level of Cu*GSS* gene and its protein amount in H_2_O_2_-treatment and control groups, respectively. (**E**) Illustration of the potential mechanism of additive H_2_O_2_ regulating laccase activity partially through the changes of GSH metabolism-related path. *Asterisked bars* in each graph differ significantly from those unmarked (Tukey’s HSD, *P* < 0.05).

As speculated, the counter-balance between GSH and ROS is important in regulating laccase production in *C. unicolor* 8761. Previous studies have shown that H_2_O_2_, a type of ROS inducer, can stimulate fungal laccase activity (30, 54). In our study, we observed that laccase production increased with increasing concentrations of additive H_2_O_2_, but excessive supplementation of H_2_O_2_ did not further enhance laccase activity (Fig. 7A). High concentration of H_2_O_2_ can lead to excessive ROS accumulation and thus cell death (55–57), which might affect laccase production by fungal cells. We also found that an acceptable concentration of H_2_O_2_ (5 mM) slightly reduced the abundance of GSH (by 4%), but significantly increased the abundance of GSSG (by 71%) (Fig. 7B). As a result, the GSH/GSSG ratio in H_2_O_2_-treated group was 45% lower than that in the control group (Fig. 7B). Furthermore, the transcription levels of Cu*Lac2*,*13*,*15*,*16* were up-regulated in the H_2_O_2_-treated group (Fig. 7C). It is important to note that not all laccase genes were activated by H_2_O_2_. For example, the transcription levels of *lcc1–3* were not affected by H_2_O_2_ (58). Additionally, Cu*Lac* genes affected by H_2_O_2_ treatment were different from those affected by APR-246-treatment (Fig. 6C; Fig. 7C). Among them, only Cu*Lac2* and Cu*Lac13* were up-regulated by both H_2_O_2_ and APR-246, while Cu*Lac3*,*5*,*9*,*10* were regulated in the opposite direction by H_2_O_2_ and APR-246. These findings suggest that the GSH metabolism pathway and H_2_O_2_-induced ROS might have each independent action mode on the regulation of laccase expression, except for the common route via regulating redox homeostasis in *C. unicolor* 87613. In the case of GSH, for instance, it is known to regulate cell proliferation and protein modification as well (38). These cellular processes might also contribute to the facilitation of Cu*Lac*s expression, which requires further experimental evidence.

Moreover, the reduction of GSH abundance induced by H_2_O_2_ also had a minor effect on the expression of Cu*GSS* gene and the amount of CuGSS enzyme (Fig. 7D). In summary, H_2_O_2_ can promote Cu*Lac2*,*13* expression through a GSH-dependent mechanism, while other unknown mechanisms might regulate the expression of the remaining Cu*Lac* genes (Fig. 7E).

### Conclusion

This study utilized transcriptome and metabolome to develop a new strategy for enhancing fungal laccase production. Through the omics analysis, we specifically targeted the glutathione metabolism pathway and its key components (GSH and Cu*GSS* gene). By manipulating the intracellular GSH levels, we demonstrated that GSH metabolism negatively regulates laccase production in *C. unicolor* 87613. To the best of our knowledge, this is the first study to uncover the impact of GSH metabolism on laccase activity. These findings provide a promising framework for improving laccase production by potentially reprogramming glutathione metabolism at a specific stage of fungal cultivation.

## MATERIALS AND METHODS

### Strains and culture conditions

The strain *C. unicolor* 87613 was obtained from the China Forestry Culture Collection Center and stored at the Key Laboratory of Marine Enzyme Engineering of Fujian Province, Fuzhou University. The strain was previously identified to be *C. unicolor* according to the sequence analysis of the *18S rRNA* gene. Its genome sequence is publicly published in the NCBI database (NCBI SRA: SRR23097119) and studied (30). The strain was revived on Potato Dextrose Agar media (PDA solid media) and incubated statically for 4–5 days at 30°C. Thereafter, three culture plugs (5 mm diameter) were cut from the revived plate using a cork borer, followed by submerged incubation in PDA liquid media at 200 rpm and 30°C.

### Assaying for extracellular laccase activity produced by *C. unicolor* 87613

The extracellular laccase (laccase) activity of *C. unicolor* 87613 was measured using 1 mL of the cultivation supernatant. According to a previous study (59), the laccase production was determined by the enzymatic oxidation rate of 2,2′-azino-bis-(3-ethylbenzothiazoline-6-sulfonic acid) (ABTS, Sigma-Aldrich, St. Louis, MO, USA). Briefly, a 2-mL reaction system containing 0.1 M pH 3.0 sodium acetate solution (975 µL), 0.5 mM ATBS (1,000 µL), and an appropriate dilution of enzyme solution (25 µL) was incubated at 45°C for 5 minutes. The optical density of the mixture (relative to the control buffer) at 420 nm was read using an ultraviolet spectrophotometer. One unit of laccase production (U) was defined as the amount of laccase required to oxidize 1 µmol ABTS in 1 minute. All assays were conducted in triplicate.

### Sequencing and analysis of *C. unicolor* 87613 transcriptome at two cultivation periods

Our earlier study showed that *C. unicolor* 87613 laccase activity peaked at cultivation day 6 (Cd-6) and then fell to a minimum at cultivation day 10 (Cd-10) (30). To explore the potential regulatory mechanism of laccase production, 0.1 g of the strain cultures at Cd-6 and Cd-10 were collected, respectively. Total RNA was extracted from three replicating cultures at Cd-6 and Cd-10 using RNAiso Reagent (TaKaRa, Dalian, China), respectively. The extracted RNA was used for library construction with the NEBNext Ultra RNA library Prep kit from Illumina. Subsequently, high-throughput sequencing was performed using the Illumina NovaSeq 6000 platform by Novogene Company (Beijing, China). Raw data (NCBI GEO: GSE236542) of fastq format were firstly processed through in-house perl script to obtain clean data, which were then mapped to the genome of *C. unicolor* 87613 (NCBI SRA: SRR23097119) (30) and normalized as fragments per kilobase of exon per million fragments mapped using Hisat2 v2.0.5 and featureCounts v1.5.0-p3, respectively. The DEGs were accepted at significant levels of |log_2_ (Cd-6/Cd-10 ratio)| ≥ 1 and of *P*-value < 0.001. Functional annotation of all DEGs was performed using non-redundant NCBI protein databases. Additionally, GO (60) and KEGG (61) analysis were carried out to classify the DEGs into three GO categories and various KEGG pathways, respectively.

### Measurement and analysis of *C. unicolor* 87613 metabolome at two cultivation periods

To gain a metabolic perspective on the regulatory mechanism of laccase activity produced by *C. unicolor* 87613, the strain was simultaneously cultivated with those for transcriptome study under the same conditions. The strain cultures at Cd-6 and Cd-10 were collected and rapidly frozen in liquid nitrogen. The frozen samples were then transported to Novogene Company (Beijing, China) using dry ice. In order to detect the profile of intracellular metabolites, an LC-MS/MS method was employed. The analysis was performed using a Vanquish UHPLC system (Thermo Fisher) coupled with an Orbitrap Q Exactive HF-X mass spectrometer (Thermo Fisher). All raw data were calculated and analyzed in accordance with previous studies (62, 63). Chemistry compounds that were mapped to at least one of the three banks (ChemSpider, mzCloud, and/or mzVault) were chosen and defined. The parameters of |log_2_ (Cd-6/Cd-10 ratio)| ≥ 1 and of *P*-value < 0.05 were used to designate the DAMs. For metabolic categorization and pathway enrichment, Metaboanalyst 5.0 (https://www.metaboanalyst.ca/) was used on all DAMs (34).

### Assaying for *C. unicolor* 87613 response to exogenous supplies

To investigate the influence of targeted glutathione metabolism on laccase activity produced by *C. unicolor* 87613, the strain was incubated for 4-day submerged cultivation at the optimal regime of 30°C and 200 rpm, followed by 2 days of additional cultivation alone (control), with 30 mM reduced glutathione (GSH), 50 µM GSH inhibitor (APR-246, equal volume of solvent DMSO was supplemented as its parallel control) (52), or 5 mM ROS inducer H_2_O_2_ (treatment). At Cd-6 period, aliquots of 0.1 g hyphal cells from the cultures were collected and ground in liquid nitrogen. Thereafter, the ground powders of each sample were suspended in 1 mL buffer 1 of Reduced or Oxidized Glutathione Assay Kits (Solarbio, Beijing, China) to evaluate the glutathione level, or in 0.1 M PBS (pH 7.4) to assay ROS level and GSH synthase content. After centrifugation at 12,000 × *g* and 4°C, the supernatants were used for each assay listed below.

### Assaying for intracellular contents of reduced and oxidized glutathione in response to exogenous supplies

The amounts of reduced glutathione (GSH) and oxidized glutathione (GSSG) in the supernatant were measured in a 5,5′-dithiobis-2-nitrobenzoic acid (DNTB) circular reaction (35, 64). A 1 mL reaction system containing sample solution (100 µL), buffer 2 (700 μL), and buffer 3 (200 μL) was prepared using a GSH Assay Kits (Solarbio, Beijing, China). The GSH contents in each system were quantified at OD_412_ after 2 minutes of standing at room temperature. For GSSG quantification, 100 µL samples were pre-treated with 2 µL buffer 2 in a GSSG Assays Kit (Solarbio, Beijing, China) to exclude GSH. After a 150-second mixed reaction of pre-treated sample (102 µL), buffer 3 (700 μL), buffer 4 (100 μL), buffer 5 (100 μL), and buffer 6 (10 μL), the GSSG contents were measured at OD_412_. The concentration of GSH or GSSG in each reaction was calculated by the GSH or GSS standard curve with sample reading at OD_412_. The final contents of GSH and GSSG were estimated as follows:


$$GSH\;or\;GSSG\;(\mu g/g\;culture)=C_{S}\times V_{S}\times N/W_{S}$$

(C*_S_* indicates the concentration of GSH or GSSG in each sample solution; V*_S_* refers to the total volume of collected supernatant (1 mL); W*_S_* stands for the weight of extracted cultures (0.1 g); N represents the dilution rate.)

### Assaying for the changes of intracellular ROS level in response to exogenous supplies

As ROS and GSH are two antagonists in balancing the intracellular redox status (41), the ROS levels in each supernatant were assessed with a ROS Assays Kit (mlbio, Shanghai, China). Briefly, each supernatant (10 µL) was diluted with 40 µL dilution buffer. Fifty microliters of blank solution, gradient-dilution standard solutions, and dilution samples were added to each well of Elisa plate following the user’s guide. Then, 100 µL of a working solution containing horseradish peroxidase (HRP) was added to each well. Following a 60-minute incubation at 37°C, the reaction mixtures were removed. A washing buffer was used to wash all reactive wells for five times. Each well was added with 50 µL substance A and B successively, before undergoing a 15-minute incubation at 37°C in the dark. The absorbance of each well at 450 nm was read after adding 50 µL terminal solution. The ROS contents in each sample were calculated according to the ROS standard curve with sample reading.

### Assaying for the altered contents of glutathione synthetase in response to exogenous supplies

Glutathione synthetase (GSS) is the last enzyme to catalyze the two-step biosynthesis of GSH (45). Therefore, GSS contents were investigated to understand the status of GSH metabolism. Based on a GSS Elisa Kit (Jingmei, Jiangsu, China), 50 µL of blank solution, gradient-dilution GSS standard solutions, and dilution samples were separately added to each well of Elisa plate, followed by a 30-minute incubation at 37°C. Subsequently, all wells were washed for 30 seconds with a washing buffer. After washing five times, 50 µL of HRP-containing buffer was added to all wells except the blank one. The incubation and washing procedures were repeated. Fifty microliters of chromogenic reagents A and B ware successively added to each well, followed by a 10-minute incubation at 37°C in the dark. The absorbance of each well was measured at 450 nm after the addition of a 50 µL terminal solution. The raw GSS contents in each sample ware determined using the GSS standard curve with sample reading at OD_450_. Additionally, protein concentration in each sample supernatant was measured with a bicinchoninic acid Protein Assay Kit (KeyGen, Nanjing, China). The final GSS contents in each sample were adjusted to an amount of nanogram per gram of protein extract (ng/g).

### Assaying for transcription pattern of laccase gene family and *GSS* in response to exogenous supplies

The precipitations of each cultivation sample were collected by centrifugation following incubation with or without exogenous supplies. Total RNAs were extracted from these precipitations and then reversed into cDNAs using a PrimeScript RT Reagent Kit (TaKaRa). Three cDNA samples of each precipitation were used as templates to quantify the transcription level of the laccase gene family and GSS by quantitative real-time PCR with paired primers (Table S1) using the TB Green Premix Ex Taq kit (TaKaRa). The reaction was operated according to the manual. Fungal 18S rRNA was used as an internal standard. The relative transcription level of each gene was calculated as the ratio of transcripts in each treatment group over that in the control group using the 2^-ΔΔCt^ method (65).

### Statistical analysis

All phenotypic parameters were quantified from the experiments with three replicates and were subjected to one-factor analysis of variance, followed by Tukey’s honestly significant difference (HSD) test for the differences of each phenotype between the control and treatment samples.

## Data Availability

The raw high throughput sequencing data of RNA sequencing have been deposited in the NCBI Sequence Read Archive (SRA) under the GEO accession number GSE236542.

## SUPPLEMENTAL MATERIAL

Fig. S1 to S2 spectrum.03405-23-s0001.pdf Two supplementary figures.

Tables S1 to S5 spectrum.03405-23-s0002.doc Five supplementary tables.

ASM does not own the copyrights to Supplemental Material that may be linked to, or accessed through, an article. The authors have granted ASM a non-exclusive, world-wide license to publish the Supplemental Material files. Please contact the corresponding author directly for reuse.
