# Supplementary material for: Transcriptomic and metabolomic analysis unveils a negative effect of glutathione metabolism on laccase activity in Cerrena unicolor 87613
Source: Microbiol Spectr. 2024 Jan 17;12(2):e03405-23. doi: 10.1128/spectrum.03405-23 (PMC10846260; doi:10.1128/spectrum.03405-23)
Supplement: Fig. S1 to S2 — Two supplementary figures. [file spectrum.03405-23-s0001.pdf]

Supplementary Figures

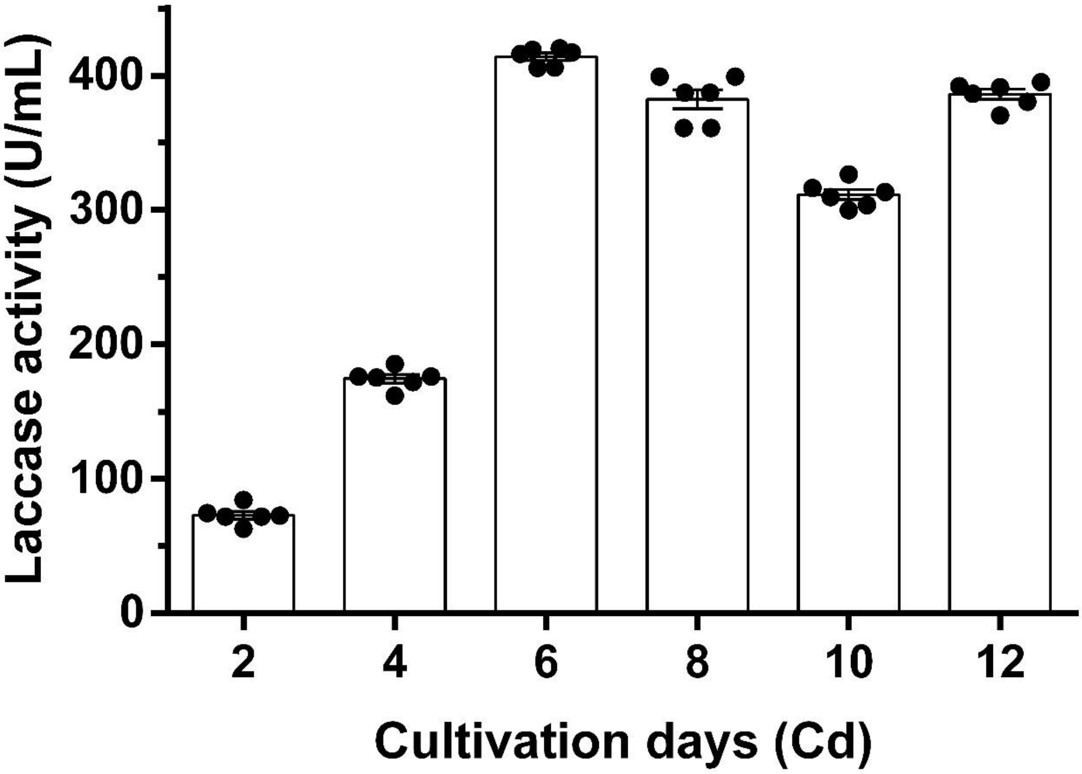

Fig S1 Laccase activity in *C. unicolor* 87613 during 12–day submerged cultivation. The maximal activity was achieved at Fd-6 with the value of 415 U/mL.

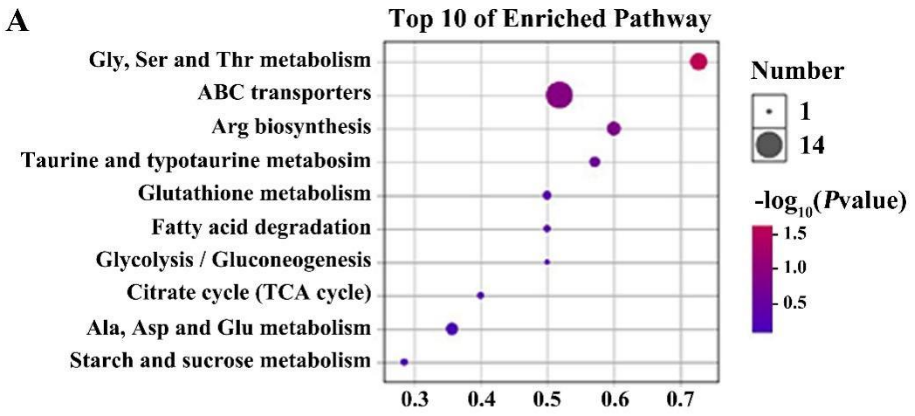

B

1. Gly, Ser and Thr metabolism

	Fd-10	Fd-6
L-Tryptophan	13	4
DL-Serine	1,727	830
Creatine	228	9
Choline	375	184
L-Threonine	6,021	2,441
Cystathionine	5,052	842
L-Serine	2,494	810

2. ABC transporters

	Fd-10	Fd-6
D-(-)-Ribose	6,821	3,174
Inositol	119	53
L-Histidine	1,773	721
Choline	375	184
L-Threonine	6,201	2,441
Biotin	7	3
Trehalose	17	7
Glutathione	28	5
L-Serine	2,494	810
L-Glutamic acid	5,554	1,594
Taurine	82	190
Glycerol-3-phosphate	380	843
Valine	4,568	9,435

3. Arginine biosynthesis

	Fd-10	Fd-6
Fumaric acid	510	168
L-Argininosuccinate	112	48
Argininosucicinic acid	1,408	536
N-Acetyl-DL-glutamic acid	124	25
L-Glutamic acid	5,554	1,594
N-Acetylornithine	229	620

4. Taurine and hypotaurine metabolism

	Fd-10	Fd-6
Acetyl phosphate	25	10
Sulfoacetic acid	72	17
L-Glutamic acid	5,554	1,594
Taurine	82	190

5. Glutathione metabolism

	Fd-10	Fd-6
L-Glutamic acid	5,554	1,594
5-oxoproline	484	213
Glutathione	28	5

6. Fatty acid degradation

	Fd-10	Fd-6
Glutaric acid	2,447	1,182
L-Palmitoylearnitine	24	116

7. Glycolysis / Gluconeogenesis

	Fd-10	Fd-6
Phosphoenolpyruvic acid	72	9

8. Citrate cycle (TCA cycle)

	Fd-10	Fd-6
Fumaric acid	510	168
Phosphoenolpyruvic acid	72	9

9. Ala, Asp and Glu metabolism

	Fd-10	Fd-6
Fumaric acid	510	168
L-Argininosuccinate	112	48
Argininosuccinic acid	1,408	536
Adenylosuccinic acid	162	33
L-Glutamic acid	5,554	1,594

10. Starch and sucrose metabolism

	Fd-10	Fd-6
Trehalose	17	7
Trhalose-6-phosphate	18	6

Increased

Decreased

Fig S2 Pathways enriched by metabolites with differential abundances. (A) The top 10 significantly enriched pathways (*P*-value < 0.05). (B) The abundance of DAMs involved in the top 10 enriched pathway ($\times10^5$). The color represented the increased (yellow) and decreased (blue) of DAMs in Cd-6 samples.
