# Supplementary material for: Transcriptomic and metabolomic analysis unveils a negative effect of glutathione metabolism on laccase activity in Cerrena unicolor 87613
Source: Microbiol Spectr. 2024 Jan 17;12(2):e03405-23. doi: 10.1128/spectrum.03405-23 (PMC10846260; doi:10.1128/spectrum.03405-23)
Supplement: Tables S1 to S5 — Five supplementary tables. [file spectrum.03405-23-s0002.doc]

**Table S1 Quality control and characterization of transcription sequencing data of *Cerrena unicolor* 87613 extracts from 6-day (Cd-6) and 10-day (Cd-10) cultivation cultures, respectively.**

| **Sample** | **Total reads** | **Clean bases**  **（bp）** | **Total mapped** | **Multiple mapped** | **Unique mapped** | **Error rate（%）** | **Q20（%）** | **Q30（%）** | **GC content（%）** |
| --- | --- | --- | --- | --- | --- | --- | --- | --- | --- |
| Cd-6-1 | 27,230,106 | 4,084,515,900 | 25,942,908  (95.27%) | 1,130,424  (4.15%) | 24,812,484  (91.12%) | 0.03 | 97.75 | 93.74 | 51.14 |
| Cd-6-2 | 26,903,658 | 4,035,548,700 | 25,621,410  (95.23%) | 1,372,623  (5.1%) | 24,248,787  (90.13%) | 0.03 | 97.72 | 93.63 | 51.01 |
| Cd-6-3 | 29,494,840 | 4,424,226,000 | 28,239,798  (95.74%) | 768,613  (2.61%) | 27,471,185  (93.14%) | 0.03 | 97.71 | 93.55 | 51.11 |
| Cd-10-1 | 30,332,250 | 4,549,837,500 | 28,938,816  (95.41%) | 1,358,492  (4.48%) | 27,580,324  (90.93%) | 0.03 | 97.6 | 93.4 | 51.07 |
| Cd-10-2 | 26,294,764 | 3,944,214,600 | 25,095,636  (95.44%) | 1,1482,26  (4.37%) | 23,947,410  (91.07%) | 0.03 | 97.65 | 93.46 | 50.96 |
| Cd-10-3 | 27,917,860 | 4,187,679,000 | 26,613,102  (95.33%) | 1,265,597  (4.53%) | 25,347,505  (90.79%) | 0.03 | 97.67 | 93.52 | 51.01 |

Total reads: the number of clean reads after quality control of sequencing data;

Clean bases: The number of bases after filtering the raw data (clean base = clean reads × 150 bp);

Total mapped: The number of reads mapped to the genome and its percentage;

Multiple mapped: The number of reads mapped to multiple locations in the reference genome and its percentage;

Unique mapped: The number of reads mapped to a unique position in the reference genome and its percentage (These data were used for subsequent quantitative data analysis of reads);

Error rate: Overall sequencing error rate;

Q20 and Q30: The percentage of bases with a Phred score greater than 20 or 30 out of the total bases, respectively;

GC content: The percentage of G and C bases in the four types of bases in clean reads.

**Table S2 Annotation of GO terms enriched by differentially expressed genes (DEGs) between Cd-6 and Cd-10 cultures.**

| **Category** | **GO ID** | **Descrption** | ***P*-value** | **Number of up/dn genes^*^** | **Rich factor^**^** |
| --- | --- | --- | --- | --- | --- |
| BP | GO：0016051 | carbohydrate biosynthetic process | 0.011 | 0 / 5 | 0.385 |
| BP | GO：0005975 | carbohydrate metabolic process | 0.000 | 4 / 37 | 0.197 |
| CC | GO：0005576 | extracellular region | 0.000 | 1 / 16 | 0.415 |
| CC | GO：0016021 | integral component of membrane | 0.010 | 22 / 31 | 0.139 |
| CC | GO：0031224 | intrinsic component of membrane | 0.010 | 22 / 31 | 0.139 |
| MF | GO：0030248 | cellulose binding | 0.000 | 0 / 13 | 0.500 |
| MF | GO：0001871 | pattern binding | 0.000 | 0 / 14 | 0.467 |
| MF | GO：0030247 | polysaccharide binding | 0.000 | 0 / 14 | 0.467 |
| MF | GO：0005507 | copper ion binding | 0.001 | 3 / 5 | 0.364 |
| MF | GO：0016701 | oxidoreductase activity | 0.011 | 0 / 5 | 0.357 |
| MF | GO：0008171 | O-methyltransferase activity | 0.008 | 1 / 5 | 0.333 |
| MF | GO：0004185 | serine-type carboxypeptidase activity | 0.015 | 5 / 0 | 0.333 |
| MF | GO：0030246 | carbohydrate binding | 0.000 | 0 / 17 | 0.315 |
| MF | GO：0016798 | hydrolase activity, acting on glycosyl bonds | 0.000 | 6 / 25 | 0.209 |
| MF | GO：0004553 | hydrolase activity, hydrolyzing O-glycosyl compounds | 0.000 | 5 / 25 | 0.207 |
| MF | GO：0016614 | oxidoreductase activity, acting on CH-OH group of donors | 0.007 | 8 / 9 | 0.195 |
| MF | GO：0048037 | cofactor binding | 0.000 | 30 / 40 | 0.162 |
| MF | GO：0020037 | heme binding | 0.007 | 12 / 22 | 0.159 |
| MF | GO：0046906 | tetrapyrrole binding | 0.007 | 12 / 22 | 0.159 |
| MF | GO：0050662 | coenzyme binding | 0.011 | 16 / 16 | 0.157 |

*up/dn genes referred to the up-regulated genes or down-regulated genes in Cd-6 cultures as compared to those in Cd-10 cultures.

**Rich factor indicated the ratio of the number of DEGs enriched in targeted pathway to the number of total annotated genes in this pathway.

**Table S3 Enrichment of KEGG terms by DEGs between Cd-6 and Cd-10 cultures.**

| **KEGG ID** | **KEGG Term** | **Gene counts^*^** | ***P*-value** | **Gene ID** | |
| --- | --- | --- | --- | --- | --- |
|  |  |  |  | **UP-regulation** | **Down-regulation** |
| tvs01110 | Biosynthesis of secondary metabolites | 50 | 0.0003 | A08064.gene/A06418.gene/A07571.gene/A06088.gene/A02716.gene/A09661.gene/A03726.gene/A00473.gene/novel.1493/A00700.gene/A06836.gene/A06660.gene/A00758.gene/A08399.gene/A00447.gene | A07715.gene/A07667.gene/A01247.gene/A03683.gene/A02991.gene/A02126.gene/A00342.gene/A00376.gene/A00533.gene/A05242.gene/A02664.gene/A04759.gene/A01721.gene/A02839.gene/A08519.gene/A02865.gene/A00980.gene/A02610.gene/A07782.gene/A06983.gene/A07134.gene/A04880.gene/A09123.gene/A01658.gene/A09404.gene/A07181.gene/A05122.gene/A03397.gene/A05128.gene/A09892.gene/A00603.gene/A05257.gene/A01289.gene/A01139.gene/A00541.gene |
| tvs01200 | Carbon metabolism | 29 | 0.0000 | A07571.gene/A04617.gene/A02716.gene/A03726.gene/novel.1493/A08399.gene | A07715.gene/A07667.gene/A01247.gene/A03683.gene/A07185.gene/A02126.gene/A00342.gene/A00533.gene/A05242.gene/A00276.gene/A04759.gene/A01721.gene/A02839.gene/A01756.gene/A09800.gene/A02610.gene/A08417.gene/A04880.gene/A07181.gene/A03397.gene/A05128.gene/A05257.gene/A00541.gene |
| tvs00500 | Starch and sucrose metabolism | 14 | 0.0001 | A09661.gene/A06836.gene | A00980.gene/A07489.gene/A07782.gene/A01792.gene/A08250.gene/A01658.gene/A01410.gene/A06736.gene/A01139.gene/A05182.gene/A05970.gene/A02880.gene |
| tvs04111 | Pyruvate metabolism | 13 | 0.0000 | A04617.gene/A03210.gene | A07715.gene/A01247.gene/A03683.gene/A03460.gene/A00342.gene/A00421.gene/A00422.gene/A04759.gene/A09800.gene/A03461.gene/A07181.gene |
| tvs00620 | Cell cycle - yeast | 13 | 0.0474 | A01254.gene/A02702.gene/A06548.gene/A06480.gene/A09761.gene/A06177.gene/A07203.gene/A09221.gene/A07582.gene/A09670.gene/A02093.gene | A01728.gene/A02602.gene |
| tvs00630 | Biosynthesis of amino acids | 12 | 0.0472 | A08064.gene/A06418.gene/A07571.gene/A02716.gene/A03726.gene/A06660.gene/A00758.gene/A08399.gene | A02664.gene/A01721.gene/A02865.gene/A04880.gene |
| tvs00010 | Glutathione metabolism | 10 | 0.0005 | A00358.gene/A09881.gene/A04379.gene/novel.1493/A09094.gene/A07600.gene/A01960.gene/A04614.gene/A04373.gene/A03741.gene | None |
| tvs00020 | Glyoxylate and dicarboxylate metabolism | 10 | 0.0015 | A00447.gene | A03683.gene/A02126.gene/A00533.gene/A00276.gene/A01721.gene/A01756.gene/A07181.gene/A05128.gene/A05257.gene |
| tvs00480 | Citrate cycle (TCA cycle) | 9 | 0.0009 | None | A07715.gene/A01247.gene/A00342.gene/A05242.gene/A04759.gene/A01721.gene/A07181.gene/A03397.gene/A00541.gene |
| tvs00350 | Meiosis - yeast | 9 | 0.0002 | A01254.gene/A07141.gene/A02702.gene/A06480.gene/A09761.gene/A06177.gene/A09221.gene/A09670.gene | A02602.gene |

*Gene counts: the number of DEG sorted in the indicated category.

**Table S4 The transcript profiles of genes involved in glutathione metabolism pathway.**

| Gene description | Gene ID | log_2_(Fold Change) | *P* value | FPKM value | |
| --- | --- | --- | --- | --- | --- |
|  |  |  |  | 6-day | 10-day |
| Aminopeptidase | A04614 | -1.02 | 0.0000 | 34.38 | 16.86 |
| γ-Glutamylcysteine | A03741 | -1.20 | 0.0001 | 123.98 | 53.75 |
| Glutathione synthetase | A09094 | -1.31 | 0.0000 | 32.12 | 12.87 |
| Glucose-6-phosphate dehydrogenase | Novel.1493 | -1.37 | 0.0000 | 174.58 | 67.15 |
| Trypanothione reductase | A07600 | -1.97 | 0.0000 | 155.65 | 39.63 |
|  | A00119 | -1.12 | 0.0014 | 52.57 | 24.15 |
| Glutathione S-transferase | A00358 | -1.68 | 0.0000 | 54.99 | 17.04 |
|  | A09881 | -1.90 | 0.0000 | 209.58 | 56.12 |
|  | A04379 | -2.51 | 0.0000 | 3523.84 | 616.81 |
|  | A04373 | -1.58 | 0.0001 | 356.42 | 118.72 |
|  | A01960 | -1.40 | 0.0000 | 59.54 | 22.52 |

**Table S5 Paired primers used for transcription profiling of laccase gene family (Cu*Lac*) and GSH synthetase gene (Cu*GSS*) in *C.unicolor* 87613.**

| **Primers** | **Sequences (5’ → 3’)** |
| --- | --- |
| Cu*Lac1-*F / R | TTACCTTCTCCATTGATG / GTCCAGTGTTGTTGTATT |
| Cu*Lac2-*F / R | CCGTTGATGCTGCGAATG / TTGTAAGTAAGGAGTGTGAGGTAG |
| Cu*Lac3-*F / R | CATCTCTTGGTCTTGTCT / TACTCTGTAGGCATTGTG |
| Cu*Lac4-*F / R | AGGTCAACGATATTCATT / GTAATATAGCGGAGTTCA |
| Cu*Lac5-*F / R | CCTGGAGCACTTATCACT / ACATACTGGCATCGGTAA |
| Cu*Lac6-*F / R | TAGGATGGATAGATGTGAAC / CAATATGGCGAGACTGTA |
| Cu*Lac7-*F / R | GGTCCTCTTGTTGTGTAT / GGTGATAACGGTAGTCTC |
| Cu*Lac8-*F / R | TATGACCATTATTGAAGTTG / ACGAATCCAGTAGTTATC |
| Cu*Lac9-*F / R | TTGAAGTGGATAGTGTTA / TTGATACCATTGTTGAAG |
| Cu*Lac10-*F / R | CTCTGTCATTAGCGTTCA / TCATATTATGGTTGTCAATC |
| Cu*Lac11-*F / R | TCGTGCTCTTAGTGCTTA / TCCGTGGAGATATTGGTATT |
| Cu*Lac12-*F / R | GAATCCACTGTTATCACCTT / TTGCGACCTAATCCATTG |
| Cu*Lac13-*F / R | CGAAGTTGATGGTGTTAA / ACGAATCCAGTAATTGTC |
| Cu*Lac14-*F / R | TTGCTGGTGGTATCAATTC / TGGTCTGCGAAGTAGTAG |
| Cu*Lac15-*F / R | CATTGAAGTTGATGGTGTT / ACGAATCCAGTAGTTGTC |
| Cu*Lac16-*F / R | AACACCAATATGACGAAGAAT / GGCAACACTGATGAACTG |
| Cu*Lac17*/18*-*F / R | GTCCTCGTCCATACCAAT / ATAAGTGAACATATCCGTCTG |
| Cu*GSS-*F / R | CTGAAGGCTTGACAGAGGC / CGACATGTGCGGATTTAGCC |
| *18S*-F / R | AGACGGAAGTTTGAGGCA / CTTCCGGCCAAGGTGAA |
